# Adjuvant olaparib in the subset of patients from Japan with *BRCA1*- or *BRCA2*-mutated high-risk early breast cancer from the phase 3 OlympiA trial

**DOI:** 10.1007/s12282-023-01451-8

**Published:** 2023-04-01

**Authors:** Hideko Yamauchi, Masakazu Toi, Shin Takayama, Seigo Nakamura, Toshimi Takano, Karen Cui, Christine Campbell, Liesbet De Vos, Charles Geyer, Andrew Tutt

**Affiliations:** 1 Japan Breast Cancer Research Group (JBCRG), Tokyo, Japan; 2 St. Luke’s International Hospital, Tokyo, Japan; 3 Kyoto University Hospital, Kyoto, Japan; 4 National Cancer Center Hospital, Tokyo, Japan; 5 Showa University Hospital, Tokyo, Japan; 6 The Cancer Institute Hospital of Japanese Foundation for Cancer Research (JFCR), Tokyo, Japan; 7 AstraZeneca, Gaithersburg, MD USA; 8 Frontier Science (Scotland) Ltd, Kingussie, UK; 9 Breast International Group (BIG), Brussels, Belgium; 10 NSABP Foundation/NRG Oncology, Pittsburgh, PA USA; 11 University of Pittsburgh Hillman Cancer Center, Pittsburgh, PA USA; 12 The Breast Cancer Now Toby Robins Research Centre, The Institute of Cancer Research, and The Breast Cancer Now Unit, Guy’s Hospital Cancer Centre, King’s College London, London, UK

**Keywords:** Adjuvant, Early breast cancer, BRCA, Olaparib, PARP inhibitor

## Abstract

**Background:**

The efficacy and safety of olaparib compared with placebo in the subset of patients from Japan in the phase 3 OlympiA trial (NCT02032823) are reported here and contextualized with reference to the global OlympiA population.

**Methods:**

Patients with germline *BRCA1* and/or *BRCA2* pathogenic variants and HER2-negative, high-risk early breast cancer who had received neoadjuvant or adjuvant chemotherapy and completed local treatment were eligible. Patients were randomized 1:1 to receive olaparib or placebo for 1 year. Primary endpoint: invasive disease-free survival (IDFS). Secondary endpoints: distant disease-free survival (DDFS), overall survival (OS), and safety. Data are reported from the first pre-specified interim analysis (data cut-off [DCO] March 27, 2020) and the second, event driven, pre-specified interim analysis of OS (DCO July 12, 2021) in patients from Japan.

**Results:**

140 patients were randomized in Japan (olaparib, n = 64; placebo, n = 76). At the first pre-specified interim analysis (median follow-up: 2.9 years), hazard ratios (HRs) for adjuvant olaparib compared with placebo were 0.5 for IDFS (95% confidence interval [CI] 0.18–1.24) and 0.41 for DDFS (95% CI 0.11–1.16). At the second pre-specified interim analysis of OS, three deaths occurred in the olaparib group versus six deaths in the placebo group (HR, 0.62 [95% CI 0.13–2.36]). Findings were consistent with those for the global population. No new safety signals were observed.

**Conclusions:**

While this analysis in a Japanese subset of patients was not powered to detect population-related treatment differences, efficacy and safety analysis results were consistent with the global OlympiA population, suggesting the findings from the global study are generalizable to clinical practice in Japan.

**Supplementary Information:**

The online version contains supplementary material available at 10.1007/s12282-023-01451-8.

## Introduction

Olaparib is an inhibitor of the poly (adenosine diphosphate–ribose) polymerase (PARP) family of enzymes, which exploits the principle of synthetic lethality to selectively kill tumor cells deficient in homologous recombination repair pathways, such as those harboring loss-of-function mutations in *BRCA1* and/or *BRCA2* genes [1, 2]. Olaparib has been extensively studied and is widely approved for use in cancers where homologous recombination repair deficiencies are common, including metastatic breast cancer (mBC) or early breast cancer (eBC) [3, 4].

The multinational phase 3 OlympiA trial compared 1 year of adjuvant olaparib with placebo in patients with germline *BRCA1* and/or *BRCA2* pathogenic variants (g*BRCA1/2*pv) and human epidermal growth factor receptor 2 (HER2)-negative, high-risk eBC. The first pre-specified interim analysis (data cut-off [DCO] March 27, 2020) demonstrated that olaparib clinically and significantly prolonged invasive disease-free survival (IDFS) and distant disease-free survival (DDFS) compared with placebo with no new safety signals [4]. While olaparib was associated with fewer deaths than placebo at the time of the first interim analysis, the between-group difference did not meet the pre-specified boundary for statistically significant differences in overall survival (OS) (p < 0.01) [4]. However, in a subsequent event-driven second interim analysis when 330 IDFS events had been reported (DCO July 12, 2021), adjuvant olaparib was associated with a statistically significant and clinically meaningful OS improvement compared with placebo (hazard ratio, 0.68; 98.5% CI 0.47–0.97; *p* = 0.009) [5]. Updated analyses of event-free rates at this second pre-specified interim analysis continued to show improvements for olaparib versus placebo; 4-year IDFS was 83% for olaparib versus 75% for placebo, and 4-year DDFS was 87 versus 79%, respectively [5].

Results from the OlympiA study led to the approval of olaparib for the adjuvant treatment of adult patients with germline *BRCA-*mutated, HER2-negative, high-risk eBC who received prior neoadjuvant or adjuvant chemotherapy ([N]ACT) in multiple countries.

Here, we report the efficacy and safety of olaparib compared with placebo in the subset of patients from Japan and contextualize the findings with reference to the outcomes in the global OlympiA population.

## Methods

### Study design

Key aspects of the methodology used in the OlympiA clinical trial (NCT02032823) will be summarized here, having been described in detail previously [4].

OlympiA is an ongoing, prospective, multicenter, multinational, double-blind, randomized, phase 3 clinical trial with an expected overall follow-up of 10 years. Enrolled patients had g*BRCA1/2*pv determined by local or central testing, with HER2-negative, hormone receptor-positive or negative, high-risk eBC. Patients were required to have completed definitive local treatment, including radiotherapy if indicated, at least 2 weeks before trial entry and to have received at least six cycles of (N)ACT containing anthracyclines, taxanes, or a combination of both. Previous platinum-based chemotherapy (CT) treatment for prior cancer, including eBC, was also permitted but was not a requirement for eligibility. Adjuvant endocrine therapy for patients with hormone receptor-positive eBC was to be administered according to local guidelines during the study treatment. Adjuvant bisphosphonates and denosumab were allowed concurrently with olaparib treatment according to investigator practice. ACT after surgery was not allowed in patients who received NACT [4].

Patients with hormone receptor-positive eBC were considered high risk if they had at least four pathologically confirmed positive lymph nodes at initial surgery prior to ACT or had evidence of lack of pathological complete response (non-pCR) to NACT with a score of at least 3 for the CPS + EG staging system [4], which estimates the probability of relapse following NACT based on baseline clinical and post-NACT pathological stages, estrogen receptor status, and nuclear grade (scores range from 0 to 6, with higher scores indicating worse prognosis) [6]. Patients with early triple-negative breast cancer (TNBC) were considered high risk if they were axillary node-positive or axillary node-negative with an invasive primary tumor measuring at least 2 cm at initial surgery prior to ACT or had a non-pCR following NACT [4].

### Treatment and assessments

Patients were randomized in a 1:1 ratio to receive olaparib 300 mg or matching placebo tablets, taken orally twice daily for 1 year. Patients were stratified according to hormone receptor status (positive or negative), timing of prior CT (NACT or ACT), and prior use of platinum-based CT (yes or no) [4].

Following randomization, patients were assessed for disease recurrence through physical examinations and medical history every 4 weeks for 24 weeks, then every 3 months through year 2, every 6 months in years 3–5, and annually after that. Breast imaging was performed every 12 months, with other imaging investigations performed at the investigator’s discretion when symptoms, physical examination findings, or laboratory results suggested the possibility of disease recurrence [4].

### Outcomes

The primary endpoint of the OlympiA trial was IDFS, defined as the time from randomization until the date of the first occurrence of one of the following events: ipsilateral invasive breast tumor, locoregional invasive disease, distant recurrence, contralateral invasive breast cancer (BC), second primary invasive cancer, or death from any cause [4, 7]. Secondary endpoints included DDFS (defined as the time from randomization until documented evidence of the first distant recurrence of breast cancer or death) and OS (defined as the time from randomization until death from any cause [4, 7]). Safety outcomes were also investigated as a secondary endpoint, assessed using the Common Terminology Criteria for Adverse Events (CTCAE), with adverse events (AEs) of special interest comprising pneumonitis, radiation pneumonitis, myelodysplastic syndrome (MDS), acute myeloid leukemia (AML), and new primary cancer other than MDS or AML [4].

An independent external data and safety monitoring committee reviewed the results of the planned first interim analysis of the primary endpoint (IDFS). They recommended proceeding with the analysis and reporting [4].

### Statistical analyses

Efficacy analyses were based on the intention-to-treat population, including all patients who underwent randomization. Survival functions were estimated utilizing Kaplan–Meier curves. The stratified Cox proportional hazards model was used to estimate hazard ratio (HRs) and confidence intervals (CIs), and a comparison of survival between treatment groups was performed using the stratified log-rank test. Safety was assessed in the population of patients who received at least one dose of olaparib or placebo [4].

Analysis of the subset of patients from Japan was performed using the global OlympiA first pre-specified interim analysis DCO date of March 27, 2020. Results for primary and key secondary endpoints of IDFS and DDFS, respectively, are presented for the subset of patients from Japan using this data. Following the event-driven pre-specified second interim analysis of OS for the global OlympiA population at DCO on July 12, 2021, the results of OS for the subset of patients from Japan are also presented here, as well as 4-year data for the primary and key secondary endpoints. Safety data are presented using data from the pre-specified second interim analysis. All data from the subset of patients from Japan is descriptive. The study was not powered to detect treatment differences in the subset of patients from Japan at either DCO.

## Results

### Patient disposition

In total, 1836 patients were randomized from June 2014 through to May 2019 to the global OlympiA trial [4]. Of these patients, 140 were randomized in Japan, with 64 to receive olaparib and 76 to receive placebo, all of whom received the assigned study treatment. During screening for the global OlympiA population, 10,514 patients underwent prospective g*BRCA* central testing, of whom 1383 (13.2%) had a confirmed g*BRCA1/2*pv. Of the total population that underwent central g*BRCA* testing, 1344 were from Japan, of whom 232 (17.3%) had a confirmed g*BRCA1/2*pv. At the time of the first pre-specified interim analysis, median follow-up was 2.9 years in the subset of patients from Japan and 2.5 years in the global OlympiA intention-to-treat population [4].

### Baseline demographics and disease characteristics

All patients in the subset from Japan were female, with a median age of 43 years (Table 1); 15.7% had hormone receptor-positive BC, and 84.3% had TNBC. Among the subset from Japan, g*BRCA1*pv were present in 73.4% of patients in the olaparib group and 63.2% in the placebo group, while g*BRCA2*pv were present in 26.6% and 36.8% of patients, respectively. No patients had both a g*BRCA1*pv and g*BRCA2*pv. Of patients that harbored a g*BRCA1*pv (n = 95), 7 (7.4%) had hormone receptor-positive BC and 88 (92.6%) had TNBC, while in patients with a g*BRCA2*pv (n = 45), 15 (33.3%) had hormone receptor-positive BC and 30 (66.7%) had TNBC. Across both treatment groups, 50.0% had received prior ACT, and 50.0% had prior NACT. Demographics and characteristics were generally similar between the subset of patients from Japan and the global OlympiA population, with the notable exception that substantially fewer patients in the subset from Japan (3.6%) had received prior platinum-based CT compared with the global OlympiA population (26.5%) (Table 1).

**Table 1:** Baseline demographics and disease characteristics in the subset of patients from Japan and the global OlympiA population

Parameter	Subset of patients from Japan (n = 140)		Global OlympiA population (n = 1836)	
	Olaparib (n = 64)	Placebo (n = 76)	Olaparib (n = 921)	Placebo (n = 915)
Age, years, median (IQR)	42 (37–50)	45 (36–53)	42 (36–49)	43 (36–50)
Female	64 (100)	76 (100)	919 (99.8)	911 (99.6)
Hormone receptor-positive	13 (20.3)	9 (11.8)	168 (18.2)	157 (17.2)
TNBC	51 (79.7)	67 (88.2)	751 (81.5)	758 (82.8)
g*BRCA1/2*pv gene affected^a^				
*BRCA1*	47 (73.4)	48 (63.2)	656 (71.2)	669 (73.1)
*BRCA2*	17 (26.6)	28 (36.8)	260 (28.2)	238 (26.0)
*BRCA1* and *BRCA2*	0	0	2 (0.2)	5 (0.5)
Prior neoadjuvant CT	27 (42.2)	43 (56.6)	460 (49.9)	460 (50.3)
Prior adjuvant CT	37 (57.8)	33 (43.4)	461 (50.1)	455 (49.7)
Prior platinum-based CT	1 (1.6)	4 (5.3)	247 (26.8)	239 (26.1)

Values presented are *n* (%) unless stated otherwise. Data cut-off: March 27, 2020

g*BRCA1/2*pv germline *BRCA1* and/or *BRCA2* pathogenic variant, *CT* chemotherapy, *IQR* interquartile range, *TNBC* triple-negative breast cancer

^a^There were six patients in the global OlympiA population who did not have g*BRCA1/2*pv; no patients in the subset from Japan were randomized without a g*BRCA1/2*pv

### Clinical efficacy

**Fig. 1:**
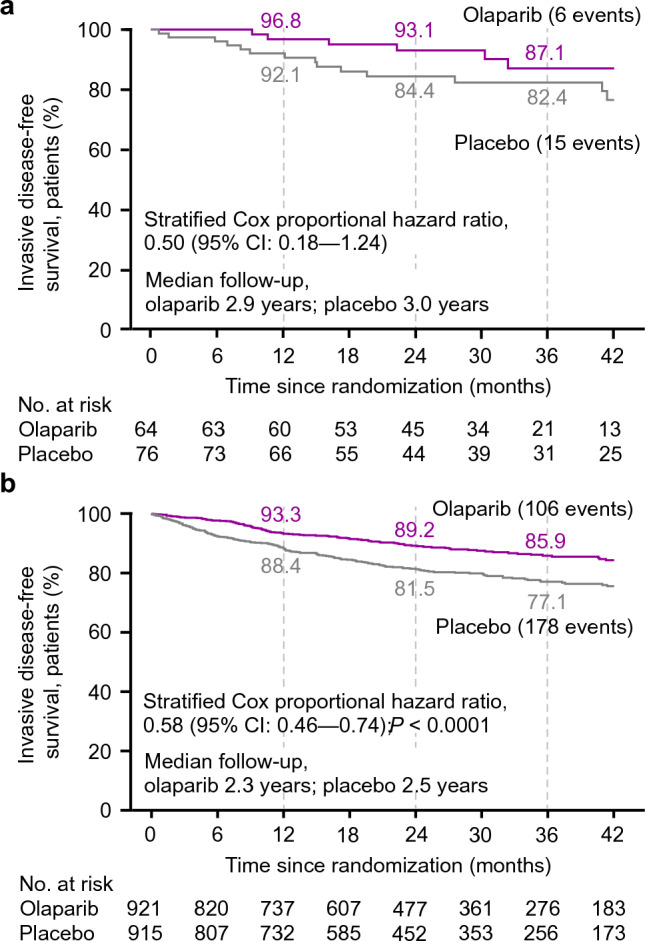
Invasive disease-free survival in the subset of patients from **a** Japan and **b** the global OlympiA population. IDFS at first pre-specified interim analysis (data cut-off March 27, 2020) in the subset of patients from Japan (panel a) and in the global OlympiA population (panel b). IDFS was defined as the time from randomization until the date of one of the following events: ipsilateral invasive breast tumor, locoregional invasive disease, distant recurrence, contralateral invasive breast cancer, second primary invasive cancer, or death from any cause. Data for patients without a documented event of invasive disease or death were censored at the date they were last known to be disease-free. Panel b From New England Journal of Medicine. Adjuvant olaparib for patients with BRCA1- or BRCA2-mutated breast cancer. Tutt ANJ, Garber JE, Kaufman B, Viale G, Fumagalli D, Rastogi P, et al. Volume 384., Page No 2399. Copyright © (2021) Massachusetts Medical Society. Reprinted with permission. *CI* confidence interval, *HR* hazard ratio, *IDFS* invasive disease-free survival, *No.*, number

At the time of the first pre-specified interim analysis, the HR for IDFS in the olaparib group (6 events) compared with the placebo group (15 events) was 0.50 (95% CI 0.18–1.24) in the subset of patients from Japan (Fig. 1a). These findings were consistent with those in the global OlympiA population, for which a 42% reduction in the risk of invasive disease recurrence or death was observed for olaparib (106 IDFS events) compared with placebo (178 IDFS events) (HR, 0.58 [95% CI 0.46–0.74; *p* < 0.0001]) (Fig. 1b). In the subset of patients from Japan, 87.1% (95% CI 72.5–94.2%) of patients in the olaparib group were alive and free of invasive disease at 3 years compared with 82.4% (95% CI 70.9–89.7%) of patients in the placebo group; this was consistent with the global OlympiA population, in which 85.9% (95% CI 82.8–88.4%) and 77.1% (95% CI 73.7–80.1%) of the olaparib group and the placebo group, respectively, were alive and free of invasive disease at 3 years. Analysis of IDFS at the time of the pre-specified second interim analysis for the subset of patients from Japan showed similar results to the first pre-specified interim analysis (Supplemental Table 1). At this time, 4-year IDFS was 86.2% (95% CI 74.1–92.9%) for patients in the olaparib group compared to 78.9% (95% CI 67.2–86.8%) for patients in the placebo group; consistent with the global OlympiA population [5].

**Fig. 2:**
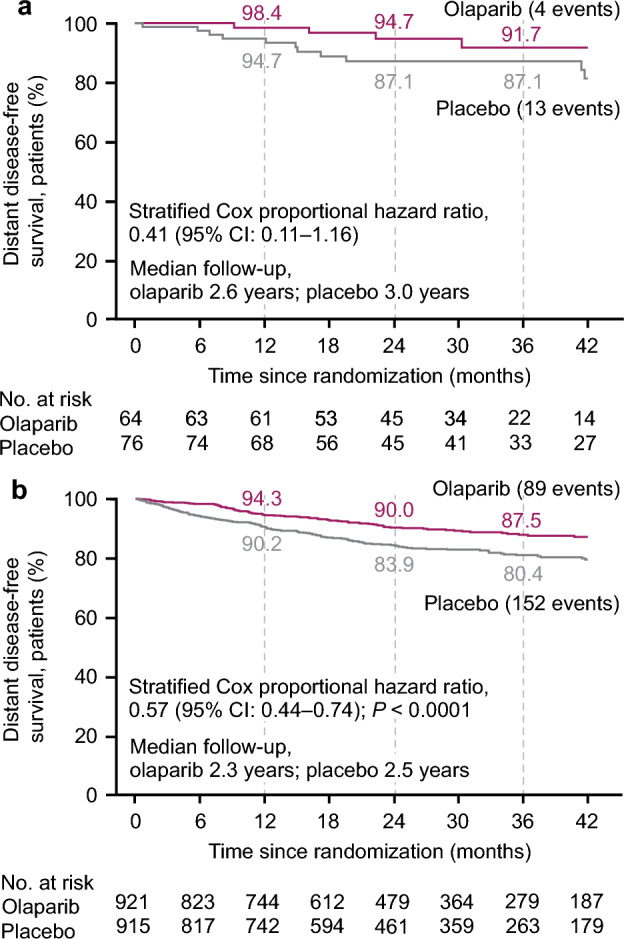
Distant disease-free survival in the subset of patients from **a** Japan and **b** the global OlympiA population. DDFS at first pre-specified interim analysis (data cut-off March 27, 2020) in the subset of patients from Japan (panel a) and in the global OlympiA population (panel b). DDFS was defined as the time from randomization until documented evidence of the first distant recurrence of breast cancer or death. Distant recurrence includes the following events: metastatic breast cancer; death attributable to any cause, including breast cancer, non-breast cancer, or unknown cause; and second primary non-breast invasive cancer. Evidence of distant recurrence requires either radiologic examination or histopathological confirmation by biopsy. Panel b From New England Journal of Medicine. Adjuvant olaparib for patients with BRCA1- or BRCA2-mutated breast cancer. Tutt ANJ, Garber JE, Kaufman B, Viale G, Fumagalli D, Rastogi P, et al. Volume 384., Page No 2399. Copyright © (2021) Massachusetts Medical Society. Reprinted with permission. *CI* confidence interval, *DDFS* distant disease-free survival, *HR* hazard ratio, *No.* number

At the time of the first pre-specified interim analysis, the HR for DDFS in the olaparib group (4 DDFS events) compared with the placebo group (13 DDFS events) was 0.41 (95% CI 0.11–1.16) in the subset of patients from Japan (Fig. 2a). This was consistent with the global OlympiA population, in which the risk of distant disease or death was reduced by 43% in the olaparib group (89 events) compared with the placebo group (152 events) (HR, 0.57 [95% CI 0.44–0.74; *p* < 0.0001]) (Fig. 2b). The observed 3-year DDFS rate in the subset of patients from Japan was 91.7% (95% CI 78.7–96.9%) in the olaparib group compared with 87.1% (95% CI 76.5–93.1%) in the placebo group, consistent with the global OlympiA population in which the 3-year DDFS rate was 87.5% (95% CI 84.6–89.9%) in the olaparib group and 80.4% (95% CI 77.2–83.3%) in the placebo group. Analysis of DDFS at the time of the pre-specified second interim analysis showed similar results (Supplemental Table 1). At the time of the second interim analysis, 4-year DDFS was 87.8% (95% CI 76.0–94.0%) in the olaparib group compared to 81.9% (95% CI 69.9–89.5%) in the placebo group in the subset of patients from Japan; consistent with the global OlympiA population [5].

At the time of the first pre-specified interim analysis, seven deaths (5.0%) had been reported in patients from Japan, three in the olaparib group and four in the placebo group (HR, 0.94 [95% CI 0.19–4.28]). At the subsequent pre-specified second interim analysis, the median OS follow-up time in the subgroup of patients from Japan was 3.9 and 4.2 years for the olaparib group and placebo group, respectively. At this time, nine deaths (6.4%) had been reported, three in the olaparib group and six in the placebo group (HR, 0.62 [95% CI 0.13–2.36]). At 4 years from randomization, the percentage of patients alive was 94.6% (95% CI 84.0–98.3%) in the olaparib group and 91.8% (95% CI 82.5–96.2%) in the placebo group. These findings are consistent with results from the global OlympiA population, where olaparib significantly improved OS compared to placebo in the second interim analysis; HR, 0.68 (98.5% CI 0.47–0.97; *p* = 0.009) [5].

### Safety

All 140 patients from Japan were included in the safety analysis (DCO July 12, 2021). The median actual treatment exposure was 353.0 days in the olaparib group and 364.0 days in the placebo group, while the median relative dose intensity was 98.9 and 100%, respectively. The median percentage of the intended dose received was 95.9% for olaparib and 99.0% for placebo.

The overall safety reported in the subset of patients from Japan was consistent with the global OlympiA population (Table 2). Most AEs were mild or moderate in severity (Grade ≤ 2). The most frequently reported Grade 3 or higher AEs in the olaparib group of the subset of patients from Japan were anemia (14.1%), neutrophil count decreased (10.9%), and white blood cell count decreased (7.8%), comparable to the global OlympiA population, in which the most frequently reported Grade 3 or higher in the olaparib group were anemia (8.7%), neutrophil count decreased (4.9%), and white blood cell count decreased (3.0%) (Table 3).

**Table 2:** Summary of safety in the subset of patients from Japan and the global OlympiA population

	Subset of patients from Japan (n = 140)		Global OlympiA population (n = 1815)	
	Olaparib (n = 64)	Placebo (n = 76)	Olaparib (n = 911)	Placebo (n = 904)
Patients with				
Any AE	64 (100)	69 (90.8)	836 (91.8)	758 (83.8)
Grade ≥ 3 AE	22 (34.4)	4 (5.3)	223 (24.5)	102 (11.3)
Serious AE	4 (6.3)	4 (5.3)	79 (8.7)	78 (8.6)
AEs leading to				
Treatment discontinuation	7 (10.9)	1 (1.3)	98 (10.8)	42 (4.6)
Dose reductions^a^	18 (28.1)	1 (1.3)	213 (23.4)	33 (3.7)
Dose interruptions^a^	26 (40.6)	8 (10.5)	286 (31.4)	99 (11.0)
Relative dose intensity,^b^ median (IQR)	98.9 (82–100)	100 (98–100)	99.5 (87–100)	100 (97–100)

Values presented are in *n* (%) unless stated otherwise. Data cut-off: July 12, 2021

*AE* adverse event, *IQR* interquartile range

^a^Includes AEs that led to dose reduction or interruption but not permanent discontinuation of study treatment

^b^Percentage of the actual total dose delivered relative to the intended total dose

**Table 3:** AEs in the subset of patients from Japan and the global OlympiA population, by preferred term and maximum reported grade (safety analysis set)

	Subset of patients from Japan (n = 140)				Global OlympiA population (n = 1815)			
	Olaparib (n = 64)		Placebo (n = 76)		Olaparib (n = 911)		Placebo (n = 904)	
	All Grade AEs	Grade ≥ 3 AEs^a^	All Grade AEs	Grade ≥ 3 AEs	All Grade AEs	Grade ≥ 3 AEs^b^	All Grade AEs	Grade ≥ 3 AEs
Patients with								
Nausea	41 (64.1)	0	18 (23.7)	0	520 (57.1)	7 (0.8)	213 (23.6)	0
Vomiting	23 (35.9)	0	7 (9.2)	0	206 (22.6)	6 (0.7)	74 (8.2)	0
Anemia	29 (45.3)	9 (14.1)	1 (1.3)	0	215 (23.6)	79 (8.7)	35 (3.9)	3 (0.3)
White blood cell count decreased	23 (35.9)	5 (7.8)	10 (13.2)	0	144 (15.8)	27 (3.0)	52 (5.8)	3 (0.3)
Neutrophil count decreased	21 (32.8)	7 (10.9)	8 (10.5)	0	147 (16.1)	45 (4.9)	59 (6.5)	7 (0.8)
Dysgeusia	14 (21.9)	0	4 (5.3)	0	107 (11.7)	0	38 (4.2)	0
Stomatitis	14 (21.9)	0	1 (1.3)	0	81 (8.9)	0	36 (4.0)	0
Fatigue	13 (20.3)	0	4 (5.3)	0	367 (40.3)	16 (1.8)	248 (27.4)	6 (0.7)
Headache	12 (18.8)	0	8 (10.5)	0	180 (19.8)	2 (0.2)	152 (16.8)	1 (0.1)
Nasopharyngitis	11 (17.2)	0	21 (27.6)	0	31 (3.4)	0	52 (5.8)	0
Diarrhea	9 (14.1)	0	12 (15.8)	0	160 (17.6)	3 (0.3)	124 (13.7)	3 (0.3)
Upper respiratory tract infection	8 (12.5)	0	12 (15.8)	0	79 (8.7)	0	75 (8.3)	2 (0.2)
Malaise	7 (10.9)	0	8 (10.5)	0	22 (2.4)	0	10 (1.1)	0
Pyrexia	7 (10.9)	0	3 (3.9)	0	48 (5.3)	1 (0.1)	41 (4.5)	1 (0.1)
Decreased appetite	7 (10.9)	1 (1.6)	2 (2.6)	0	119 (13.1)	2 (0.2)	53 (5.9)	0
Dizziness	4 (6.3)	0	2 (2.6)	0	104 (11.4)	1 (0.1)	66 (7.3)	1 (0.1)
Arthralgia	2 (3.1)	1 (1.6)	5 (6.6)	0	89 (9.8)	2 (0.2)	115 (12.7)	2 (0.2)

Values presented are *n* (%). Data cut-off: July 12, 2021. Shown are AEs of any grade with an incidence of at least 10% in either treatment group in the safety analysis set from the subset of patients from Japan or the global OlympiA population

*AE* adverse event

^a^All listed AEs from the subset of patients from Japan are Grade 3 except for two Grade 4 events in the olaparib group involving decreased neutrophil count

^b^All listed AEs from the global OlympiA population are Grade 3 except for 10 Grade 4 events in the olaparib group: five events involving decreased neutrophil count, four involving anemia, and one involving fatigue

Serious AEs were reported at a similar rate between the olaparib group (6.3%) and the placebo group (5.3%) in the subset from Japan, rates comparable to the global OlympiA population (Table 2).

Among the subset of patients from Japan, none in the olaparib group and one patient in the placebo group (1.3%) had developed AML; in the global OlympiA population, two (0.2%) and three (0.3%) patients had developed AML in the olaparib group and placebo group, respectively. None of the patients from Japan in the olaparib group received a blood transfusion. In contrast, one patient (0.7%) in the placebo group received multiple blood products as part of management for AML.

Among the patients from Japan, four (6.3%; breast cancer [n = 2], colorectal cancer, and gastric cancer) patients in the olaparib group and three (3.9%; malignant lung neoplasm, fallopian tube cancer and transitional cell carcinoma) patients in the placebo group had developed new primary cancers > 30 days after finishing treatment. One patient in the placebo group also developed ovarian cancer with onset within 30 days of stopping study treatment. In the global OlympiA population, 21 (2.3%) and 36 (4.0%) patients in the olaparib- and placebo groups, respectively, had developed new primary cancers [5]. Radiation pneumonitis occurred in one patient (1.6%) from Japan in the olaparib group and two patients (2.6) from Japan in the placebo group within 30 days of stopping study treatment.

Among the subset of patients from Japan, seven patients (10.9%) in the olaparib group and one patient (1.3%) in the placebo group permanently discontinued study treatment owing to AEs, which was consistent with the observation of a higher number of permanent discontinuations due to AEs in the global OlympiA population in the olaparib arm (98 [10.8%] in the olaparib group and 42 [4.6%] in the placebo group). All deaths in patients from Japan were the results of breast cancer, except for one patient in the placebo group who died due to AML (considered an AE).

## Discussion

The present analysis was conducted to support the approval of olaparib as adjuvant therapy in *BRCA*-mutated, HER2-negative eBC at high risk of recurrence by the Japanese Pharmaceutical and Medical Devices Agency. The results of the descriptive analyses for IDFS and DDFS in the subgroup of patients from Japan were consistent with the definitive results reported in the overall study population, which demonstrated statistically significant and clinically meaningful improvements in the primary endpoint of IDFS and the secondary endpoint of DDFS for the global OlympiA population at the time of the first pre-specified interim analysis. OS results in the subset of patients from Japan at the time of the pre-specified second interim analysis were also consistent with results reported in the global OlympiA population, where statistical significance was demonstrated [5]. Consistency between data from the subset of patients from Japan and the global OlympiA population was also seen for 4-year event-free rates at this second analysis point [5].

Safety data for the subset of patients from Japan were consistent with the known safety profile of olaparib in eBC and in the treatment of other advanced or metastatic cancers [8], and no new safety signals were observed. Most AEs were mild or moderate in severity. While there was a slightly higher incidence of Grade ≥3 AEs in patients receiving olaparib in the subset from Japan than in the global OlympiA population, the number of patients requiring permanent discontinuation of treatment due to AEs was similar between populations. Hematological toxicities occurred at a slightly higher rate in the subset of patients from Japan and are also more common in Asian patients than non-Asian patients following the administration of CT and targeted therapies for breast cancer treatment [9]. Hematological AEs can generally be managed using an effective dose adjustment strategy [9], which could further reduce the already low number of patients requiring permanent discontinuation of study treatment due to AEs. AML and MDS were considered AEs of special interest in the OlympiA trial. As such, it is reassuring that no cases were reported in the olaparib group for the subset of patients from Japan, though long-term follow-up is still warranted for a complete assessment of risk. 

The OlympiA study was designed to assess the efficacy and safety of olaparib in patients with g*BRCA1/2*pv and high-risk, HER2-negative eBC, irrespective of hormone receptor status. While olaparib is the only treatment option specifically for patients with g*BRCA1/2*pv, other treatment options are available in Japan for use as adjuvant treatment in patients at high risk of recurrence. As such, physicians will need to choose between olaparib and other agents available in this patient population.

For patients with TNBC at high risk of recurrence, pembrolizumab is the only approved treatment option. Pembrolizumab can be used in combination with NACT and continued as a single-agent adjuvant treatment after surgery based on the results of the KEYNOTE-522 trial, which demonstrated a significant difference in pCR rate with the addition of pembrolizumab to NACT (64.8% [95% CI 59.9–69.5%]) compared with CT alone (51.2% [95% CI 44.1–58.3%]), and significant event-free survival (HR, 0.63 [95% CI 0.48–0.82]) [10, 11]. Assessment of the Asian subset of patients in the KEYNOTE-522 study, which included patients from Japan, Korea, Taiwan, and Singapore, demonstrated consistent findings with the overall study population [12]. However, the KEYNOTE-522 trial did not evaluate the g*BRCA1/2*pv status of participating patients; outcomes in this specific population of patients are unknown.

Reflecting the lack of clarity regarding clinical benefit of adjuvant capecitabine, treatment is not approved or reimbursed in Japan, but is recommended by the Japanese Breast Cancer Society clinical practice guidelines and Pan-Asian guidelines as a treatment option for patients with TNBC not achieving pCR following NACT [13, 14]. Data from the CREATE-X trial in patients from Japan and South Korea showed that adjuvant capecitabine demonstrated significantly longer disease-free survival (HR, 0.58 [95% CI 0.39–0.87]) and OS (HR, 0.52 [95% CI 0.30–0.90]) in patients with TNBC following standard NACT [15]. Although capecitabine has not been specifically evaluated in patients with g*BRCA1/2*pv high-risk eBC, adjuvant capecitabine has been studied in patients with basal subtype TNBC, the subtype patients with *gBRCA1/2*pv typically develop [16, 17]. In a preplanned subgroup analysis of the GEICAM/CIBOMA study, which investigated adjuvant capecitabine following standard (N)ACT in TNBC, benefit of capecitabine was observed in non-basal patients compared to patients with the basal phenotype [16]. Patients with basal subtype TNBC also have been shown to have a worse prognosis regardless of treatment with either adjuvant capecitabine or platinum chemotherapy compared to patients with nonbasal subtype in the ECOG-ACRIN EA113127 randomized trial, highlighting the need for alternative treatment approaches for patients with basal subtype TNBC [17]. Preliminary research suggests the addition of capecitabine to conventional ACT may be more effective in non-*BRCA1-*like TNBC than in *BRCA1*-like TNBC [18]. Currently, more research is needed to elucidate the benefits of capecitabine in patients with *gBRCA1/2*pv.

For patients with high-risk, hormone-receptor-positive/HER2-negative eBC, abemaciclib is a treatment option in Japan based on the results from the monarchE trial. Abemaciclib, when combined with endocrine therapy, resulted in a significant difference in IDFS compared with endocrine therapy alone (HR, 0.70 [95% CI 0.59–0.82]), although survival benefits have not yet been reported [19]. Data from patients from Japan treated in the monarchE trial has not yet been reported. However, data analysis in advanced breast cancer showed that abemaciclib, in combination with endocrine therapy in the East Asian populations, provided consistent results with the overall population [20]. It should be noted that, as in the KEYNOTE-522 trial, monarchE was not designed to assess activity in patients with g*BRCA1/2*pv, and there is evidence to suggest that hormone-receptor-positive mBC and g*BRCA2*pv may have poor outcomes when treated with cyclin-dependent kinase 4 and 6 inhibitors [21–23]. As such, the specific efficacy of abemaciclib in patients with g*BRCA1/2*pv and high-risk eBC needs to be established to support use.

Potential limitations of the present analysis are the relatively small number of patients in the subset from Japan and the fact that the analysis of this subset of patients was not pre-specified and is descriptive. The lower percentage of patients in the subset from Japan who had received prior platinum-based CT compared with the global OlympiA population should also be considered when interpreting the results. This difference likely resulted from the fact that the use of cisplatin is not covered by insurance for patients with eBC in Japan [14].

To our knowledge, this analysis is the first to assess the clinical benefit and safety of a PARP inhibitor for use as adjuvant therapy in patients with g*BRCA1/2*pv eBC treated in Japan. The consistency of results between the subset of patients from Japan and the global OlympiA population supports the clinical benefit of adjuvant olaparib for patients with g*BRCA1/2*pv and HER2-negative, high-risk eBC in Japan after completion of local treatment and neoadjuvant or adjuvant CT. The OlympiA trial is ongoing, with 10-year patient follow-up to provide descriptive efficacy and safety analyses.

## Supplementary Information

Below is the link to the electronic supplementary material.

Supplementary file1 (DOCX 79 KB)

## Data Availability

Data underlying the findings described in this manuscript may be obtained in accordance with the AstraZeneca data sharing policy, described at: https://astrazenecagrouptrials.pharmacm.com/ST/Submission/Disclosure.
