# Correction: Adjuvant olaparib in the subset of patients from Japan with BRCA1- or BRCA2-mutated high-risk early breast cancer from the phase 3 OlympiA trial

**DOI:** 10.1007/s12282-023-01468-z

**Published:** 2023-05-12

**Authors:** Hideko Yamauchi, Masakazu Toi, Shin Takayama, Seigo Nakamura, Toshimi Takano, Karen Cui, Christine Campbell, Liesbet De Vos, Charles Geyer, Andrew Tutt

**Affiliations:** 1 Japan Breast Cancer Research Group (JBCRG), Tokyo, Japan; 2 St. Luke’s International Hospital, Tokyo, Japan; 3 Kyoto University Hospital, Kyoto, Japan; 4 National Cancer Center Hospital, Tokyo, Japan; 5 Showa University Hospital, Tokyo, Japan; 6 The Cancer Institute Hospital of Japanese Foundation for Cancer Research (JFCR), Tokyo, Japan; 7 AstraZeneca, Gaithersburg, MD USA; 8 Frontier Science (Scotland) Ltd, Kingussie, UK; 9 Breast International Group (BIG), Brussels, Belgium; 10 NSABP Foundation/NRG Oncology, Pittsburgh, PA USA; 11 University of Pittsburgh Hillman Cancer Center, Pittsburgh, PA USA; 12 The Breast Cancer Now Toby Robins Research Centre, The Institute of Cancer Research, and The Breast Cancer Now Unit, Guy’s Hospital Cancer Centre, King’s College London, London, UK

**Correction: Breast Cancer** 10.1007/s12282-023-01451-8

The article “Adjuvant olaparib in the subset of patients from Japan with BRCA1‑ or BRCA2‑mutated high‑risk early breast cancer from the phase 3 OlympiA trial”, written by Hideko Yamauchi, Masakazu Toi, Shin Takayama, Seigo Nakamura, Toshimi Takano, Karen Cui, Christine Campbell, Liesbet De Vos, Charles Geyer Jr, Andrew Tutt, was originally published electronically on the publisher’s internet portal on 01 April 2023 without open access. With the author(s)’ decision to opt for Open Choice the copyright of the article changed on 20 April 2023 to © The Author(s) 2023 and the article is forthwith distributed under a Creative Commons Attribution4.0 International License, which permits use, sharing, adaptation, distribution and reproduction in any medium or format, as long as you give appropriate credit to the original author(s) and the source, provide a link to the Creative Commons licence, and indicate if changes were made. The images or other third party material in this article are included in the article's Creative Commons licence, unless indicated otherwise in a credit line to the material. If material is not included in the article's Creative Commons licence and your intended use is not permitted by statutory regulation or exceeds the permitted use, you will need to obtain permission directly from the copyright holder. To view a copy of this licence, visit http://creativecommons.org/licenses/by/4.0/.

