# Supplementary material for: Adjuvant olaparib in the subset of patients from Japan with BRCA1- or BRCA2-mutated high-risk early breast cancer from the phase 3 OlympiA trial
Source: Breast Cancer. 2023 Apr 1;30(4):596–605. doi: 10.1007/s12282-023-01451-8 (PMC10284949; doi:10.1007/s12282-023-01451-8)
Supplement: Supplementary file 1 — Supplementary file1 (DOCX 79 KB) [file 12282_2023_1451_MOESM1_ESM.docx]

## Supplemental Table 1. Invasive disease-free survival and distant disease-free survival in the subset of patients from Japan and the global OlympiA population at the time of the second interim analysis

|  | **Subset of patients from Japan**  **(*n* = 140)** | | **Global OlympiA population**  **(*n* = 1836)** | |
| --- | --- | --- | --- | --- |
|  | **Olaparib**  (*n* = 64) | **Placebo**  (*n* = 76) | **Olaparib**  (*n* = 921) | **Placebo**  (*n* = 915) |
| **IDFS** |  |  |  |  |
| Number of events (%) | 9 (14.1) | 16 (21.1) | 134 (14.5) | 207 (22.6) |
| Hazard ratio (95% CI) | 0.67 (0.28–1.48) | | 0.63 (0.50–0.78) | |
| Kaplan-Meier estimate of IDFS; % (95% CI) | | | | |
| 1-year | 96.8 (87.9–99.2) | 90.8 (81.6–95.5) | 93.4 (91.5–94.9) | 88.4 (86.1–90.3) |
| 2-year | 93.7 (84.0–97.6) | 84.2 (73.9–90.7) | 89.7 (87.4–91.6) | 81.4 (78.7–83.8) |
| 3-year | 88.3 (77.1–94.3) | 82.7 (72.1–89.6) | 86.1 (83.5–88.3) | 77.3 (74.3–80.0) |
| 4-year | 86.2 (74.1–92.9) | 78.9 (67.2–86.8) | 82.7 (79.6–85.4) | 75.4 (72.2–78.3) |
| **DDFS** |  |  |  |  |
| Number of events (%) | 7 (10.9) | 13 (17.1) | 107 (11.6) | 172 (18.8) |
| Hazard ratio (95% CI) | 0.64 (0.24–1.57) | | 0.61 (0.48–0.77) | |
| Kaplan-Meier estimate of DDFS; % (95% CI) | | | | |
| 1-year | 98.4 (89.3–99.8) | 94.7 (86.6–98.0) | 94.4 (92.6–95.7) | 90.3 (88.2–92.1) |
| 2-year | 95.2 (86.0–98.4) | 88.1 (78.4–93.6) | 90.6 (88.4–92.4) | 84.0 (81.4–86.3) |
| 3-year | 89.9 (78.9–95.4) | 88.1 (78.4–93.6) | 88.0 (85.5–90.1) | 81.0 (78.1–83.5) |
| 4-year | 87.8 (76.0–94.0) | 81.9 (69.9–89.5) | 86.5 (83.8–88.8) | 79.1 (76.0–81.8) |

Data cut-off: July 12, 2021.
*CI* confidence interval, *DDFS* distant disease-free survival, *HR* hazard ratio, *IDFS* invasive disease-free survival.
